# Beyond injury severity: monocyte-associated small extracellular vesicles distinguish respiratory-support trajectories following thoracic trauma

**DOI:** 10.1186/s12890-026-04653-w

**Published:** 2026-08-31

**Authors:** Aliona Wöhler, Arnulf G. Willms, Andreas Scholz, Nina Kühler, Robert Schwab, Paula Müller, Miroslaw T. Kornek

**Affiliations:** 1 Department of General, Visceral and Thoracic Surgery, German Armed Forces Hospital Koblenz, Rübenacher Str. 170, Koblenz, 56072 Germany; 2 Institute of Molecular Medicine and Experimental Immunology, University Hospital of the Rheinische Friedrich-Wilhelms-University, Venusberg Campus 1, Geb. 12, BMZII, Bonn, 53127 Germany

**Keywords:** Biomarker, Respiratory failure, Extracellular vesicles, Exosomes, Liquid biopsy, Thorax trauma, Tracheostomy

## Abstract

Thoracic trauma is a major contributor to morbidity in severely injured patients and is frequently associated with prolonged mechanical ventilation, tracheostomy, and extended intensive care unit (ICU) treatment. Early identification of patients at risk for unfavorable respiratory-support trajectories remains challenging. Small extracellular vesicles (sEVs) are rapidly released in response to cellular activation and injury and may provide biological information complementary to established clinical parameters. In our study we analyzed CD9⁺CD14⁺ and CD9⁺CD61⁺ sEV subpopulations in 45 patients with thoracic trauma. sEV levels were quantified within 24 h after admission and evaluated in relation to clinically relevant respiratory-support trajectories, including the predefined primary respiratory-support endpoints of tracheostomy and prolonged mechanical ventilation (> 5 days). Prolonged ICU stay (> 5 days) was analyzed as a supportive endpoint reflecting overall intensive-care burden. Predictive performance was assessed using repeated cross-validated receiver operating characteristic (ROC) analysis and compared with predefined clinical injury, laboratory, and oxygenation-based models. Residual analyses were performed to determine whether EV-associated information extended beyond established thoracic injury severity scores. CD9⁺CD14⁺ sEVs represented the dominant EV signal and were significantly elevated in patients requiring tracheostomy (3.84-fold, *p* = 0.009), prolonged mechanical ventilation > 5 days (4.88-fold, *p* = 0.003), and prolonged ICU treatment > 5 days (5.62-fold, *p* = 0.003). Within this exploratory cohort, CD9⁺CD14⁺ sEVs demonstrated performance comparable to the clinical injury model and consistently performed significantly better than laboratory- and oxygenation-based approaches across multiple respiratory-support trajectory endpoints. Sensitivity analyses using stricter thresholds (> 10 days) yielded similar findings. Residual analyses further demonstrated that patients with higher-than-expected CD9⁺CD14⁺ levels relative to AIS Chest and TTSS exhibited increased rates of tracheostomy and prolonged mechanical ventilation, suggesting that EV-associated information is not fully explained by anatomical injury severity alone. We concluded that early CD9⁺CD14⁺ sEV levels are associated with unfavorable respiratory-support trajectories following thoracic trauma. While established injury scores primarily describe the extent of anatomical damage, EV-associated biomarkers may provide complementary information regarding the biological response to injury. These findings support further investigation of EV-based biomarkers as tools for early risk stratification in thoracic trauma and warrant validation in larger independent cohorts.

**Supplementary Information:**

The online version contains supplementary material available at https://doi.org/10.1186/s12890-026-04653-w.

## Highlights

Early CD9⁺CD14⁺ sEVs identified adverse respiratory-support trajectories.

CD9⁺CD14⁺ sEVs predicted tracheostomy and prolonged ventilation.

EVs performed comparably to established clinical injury models.

Laboratory and oxygenation models showed lower predictive value.

EVs may capture biological response beyond anatomical injury burden.

**Supplementary Information:**

The online version contains supplementary material available at https://doi.org/10.1186/s12890-026-04653-w.

## Introduction

Thoracic trauma remains a significant contributor to morbidity and mortality in trauma patients worldwide, accounting for approximately one-third of all trauma-related deaths [1]. The clinical course of thoracic injury is highly heterogeneous and can range from minor rib fractures requiring analgesia alone to severe lung contusions with intensive care unit (ICU) support, prolonged need for mechanical ventilation, or even tracheostomy for weaning. Despite advances in clinical imaging, monitoring, and supportive care, early risk stratification for the severity of the trauma remains a clinical challenge. Several scoring systems have been developed in recent years to define injury severity in cases of thoracic trauma [2]. The Abbreviated Injury Scale (AIS) and the Injury Severity Score (ISS) are broad tools for assessing polytrauma, lacking in-depth injury analysis. Despite the development of various clinical prediction models for blunt chest trauma patients, there is still no universally accepted model in clinical practice. Systematic review by Battle et al. identified 22 clinical prediction models and pathways for evaluating the blunt chest trauma. While some models show acceptable predictive ability, the lack of external validation and high risk of bias restricts their widespread use [3].

Specific existing scoring systems such as the Thoracic Trauma Severity Score (TTSS), RibScore, Lung Organ Failure Score, or Tracheostomy in Thoracic Trauma Prediction Score (T3P-Score) provide useful structural overviews but lack molecular resolution and often fail to anticipate the outcome of organ dysfunction [4–7].

Polytrauma patients with thoracic trauma are at greater risk of prolonged mechanical ventilation than patients without severe thoracic injuries [8]. Other factors, such as patient comorbidity, age, or immune status, make the creating a universally accepted outcome scores extremely difficult.

Small extracellular vesicles (sEVs) have emerged as promising biomarkers in diverse inflammatory and injury contexts [9–14]. These nanosized, membrane-bound particles are actively released by most cell types under stress or activation and carry bioactive cargo such as surface markers, proteins, lipids, and RNAs reflective of their cell of origin and physiological state [15–17]. Importantly, sEVs release often precedes classical biomarker changes, making them attractive candidates for early and dynamic monitoring in acute care settings [18]. In trauma and critical care, EVs have been linked to immune modulation, endothelial dysfunction, and organ injury, but their predictive potential in well-defined trauma subtypes remains underexplored [10, 11, 18–20].

Recent work has demonstrated that surface profiling of specific EV subpopulations may uncover biological signals otherwise undetectable in bulk plasma or serum analysis. In this context, platelet-associated (CD61⁺) and monocyte-associated (CD14⁺) sEVs have been associated with injury severity and immune activation in trauma and sepsis [10–12, 21].

In this study, we sought to evaluate the utility of CD9⁺CD14⁺ and CD9⁺CD61⁺ sEVs—captured and quantified using high-resolution surface marker profiling—as predictive biomarkers in a cohort of thoracic trauma patients. We specifically investigated whether early sEV measurements are associated with subsequent respiratory-support trajectories, including tracheostomy, prolonged mechanical ventilation, and prolonged ICU treatment. To place EV-associated information into a clinically meaningful context, predictive performance was systematically compared with established clinical injury scores, routine laboratory parameters, and oxygenation-based approaches including the PaO₂/FiO₂ ratio. Furthermore, we explored whether EV-associated signals merely reflect anatomical injury severity or provide additional biological information beyond established thoracic trauma scoring systems. By integrating sEV profiling with clinical, laboratory, and physiological information layers, this exploratory study aimed to assess the potential role of EVs as complementary biomarkers for early risk stratification following thoracic trauma and to generate hypotheses for future validation studies.

## Patients and methods

### Study cohort and clinical context

This study represents a structured reanalysis of a previously published observational cohort of trauma patients (Kornek et al., Frontiers in Immunology, 2023) [10]. The original cohort comprised 63 severely injured trauma patients, of whom 45 presented with clinically confirmed thoracic trauma, including rib fractures, hemothorax, pneumothorax, pulmonary contusion, and flail chest (Table 1). All patients were treated at the German Armed Forces Central Hospital (Bundeswehrzentralkrankenhaus Koblenz, Koblenz, Germany). The Ethics Committee of the responsible State Chamber of Medicine in Rhineland-Palatinate, Germany, approved the human study (ANr.: 2020–15050). Informed consent was obtained from all patients or their legal representatives.

**Table 1:** Patient characteristics and clinical outcomes in thoracic trauma stratified by tracheostomy

Variable	Total	Tracheostomy yes	Tracheostomy no	*p*-value
Total patients	*n* = 45	*n* = 14	*n* = 31	
Gender (f/m)	10 / 35	2 / 12	8 / 23	
Age (years)	53.6 ± 16.91 (20–79)	49.9 ± 19.19 (20–77)	55.3 ± 15.82 (24–79)	0.454
BMI	27.3 ± 3.84 (20–39)	27.7 ± 2.55 (22–31)	27.1 ± 4.33 (20–39)	0.377
ISS	30.7 ± 12.40 (12–50)	39.6 ± 13.05 (18–50)	26.7 ± 9.92 (12–50)	0.005
TTSS	6.8 ± 3.57 (1–15)	9.0 ± 3.90 (4–15)	5.7 ± 2.94 (1–13)	0.010
AIS chest	2.9 ± 1.18 (1–5)	3.6 ± 1.08 (2–5)	2.6 ± 1.09 (1–5)	0.008
Rib score	1.9 ± 1.36 (0–4)	2.2 ± 1.58 (0–4)	1.8 ± 1.26 (0–4)	0.394
WBC (admission) [×10³/µL]	12.8 ± 5.72 (4–30)	13.0 ± 7.31 (4–30)	12.7 ± 4.98 (5–30)	0.864
WBC (day 1) [×10³/µL]	10.1 ± 3.71 (4–24)	9.7 ± 4.58 (5–24)	10.3 ± 3.30 (4–21)	0.284
CRP (day 1) [mg/dL]	4.2 ± 4.12 (0–13)	4.6 ± 4.60 (0–13)	4.1 ± 3.95 (0–13)	0.912
IL-6 (day 1) [pg/mL]	62.5 ± 158.58 (0-680)	112.2 ± 210.15 (0-587)	40.0 ± 126.76 (0-680)	0.390
Albumin (day 1) [g/L]	22.0 ± 14.68 (0–44)	26.2 ± 9.14 (0–41)	20.1 ± 16.43 (0–44)	0.541
Ventilation (days)	7.9 ± 12.86 (0–60)	23.1 ± 13.56 (10–60)	1.1 ± 2.66 (0–11)	< 0.001
ICU days	10.7 ± 13.44 (0–64)	26.4 ± 13.86 (11–64)	3.6 ± 3.87 (0–13)	< 0.001
ASA 1	4 (9%)	2 (14%)	2 (6%)	0.578
ASA 2	28 (62%)	6 (43%)	22 (71%)	0.101
ASA 3	13 (29%)	6 (43%)	7 (23%)	0.286
Pulmonary disease	7 (16%)	1 (7%)	6 (19%)	0.407
Pulmonary contusion	24 (53%)	10 (71.4%)	14 (45.2)	0.121
Pneumothorax	17 (38%)	9 (64%)	8 (26%)	0.021
Hemothorax	8 (18%)	3 (21%)	5 (16%)	0.689
Abdominal trauma	21 (47%)	8 (57%)	13 (42%)	0.520
Pelvic injury	11 (24%)	5 (36%)	6 (19%)	0.277
Traumatic brain injury	13 (29%)	3 (21%)	10 (32%)	0.724
Respiratory failure	19 (42%)	13 (93%)	6 (19%)	< 0.001
Early respiratory failure	18 (40%)	11 (79%)	7 (23%)	< 0.001
Delayed respiratory failure	2 (4%)	2 (14%)	0 (0%)	0.092
Pneumonia	9 (20%)	6 (43%)	3 (10%)	0.017

Demographic, clinical, and laboratory characteristics of patients with thoracic trauma treated at the German Armed Forces Central Hospital are presented for the overall cohort and stratified by tracheostomy status. Categorical variables are reported as absolute numbers (n) with percentages. Continuous variables are presented as mean ± standard deviation (SD) with corresponding ranges. Group comparisons between patients with and without tracheostomy were performed using the Mann–Whitney U test for continuous variables and Fisher’s exact test or χ² test for categorical variables, as appropriate. A two-sided p-value < 0.05 was considered statistically significant. Early respiratory failure was defined as the requirement for mechanical ventilation either prehospital or within 48 h. Delayed respiratory failure was defined as the need for mechanical ventilation occurring after hospital day 2. Due to the low number of events, delayed respiratory failure was not included in further statistical analyses

Blood samples for small extracellular vesicle (sEV) profiling were collected within the first 24 h following trauma admission. For the present analysis, only polytrauma patients with thoracic trauma and available sEV marker data were included. This sub-analysis focuses specifically on thoracic injury and associated EV dynamics, extending our previously published findings [10].

Because traumatic brain injury (TBI) may influence early airway management and mechanical ventilation independent of thoracic injury severity, additional TBI-adjusted analyses were performed for oxygenation-based models.

### Ethics

All patients were enrolled under an approved protocol from the Ethics Committee of the State Chamber of Physicians in Rhineland-Palatinate, Germany (Approval No. 2020–15050). No active German Armed Forces soldiers or soldiers from other nations were included.

Informed consent was obtained from all participants or their legal representatives in accordance with the Declaration of Helsinki. The initial study is registered in the German Clinical Trials Registry (DRKS00026025) and represents a new sub-analysis focused specifically on thoracic trauma and its associated small extracellular vesicle profiles, expanding upon findings previously published in 2023 [10].

### sEV isolation, enrichment, and surface marker profiling

The serum-based extracellular vesicle (EV) workflow used in the present study was previously established and biologically validated in our original human polytrauma cohort investigation [10]. Blood samples were collected within 24 h after trauma admission and processed according to standardized pre-analytical procedures. Following centrifugation, serum was separated, aliquoted, and stored at − 80 °C until further analysis. All samples were handled under identical conditions to minimize pre-analytical variability.

Small extracellular vesicles (sEVs) were enriched from serum using size-exclusion chromatography (SEC). Prior to SEC, serum samples were clarified to remove residual cells, cellular debris, larger vesicles, and aggregates. Subsequently, 1 mL of serum was applied to qEVoriginal SEC columns (IZON Science, Christchurch, New Zealand) pre-equilibrated with phosphate-buffered saline (PBS). EV-enriched fractions were collected according to the manufacturer’s protocol, pooled, and concentrated using centrifugal filter devices. Aliquots of the concentrated sEV preparation were stored at − 80 °C until analysis. To ensure comparability between samples, all specimens underwent identical isolation, concentration, storage, and thawing procedures.

The biological validity of the resulting sEV preparations was established in the original cohort study using multiple orthogonal characterization approaches consistent with contemporary EV research standards, including nanoparticle tracking analysis (NTA), transmission electron microscopy (TEM), and protein-based characterization of EV-associated markers [10]. As these validation experiments have been reported previously, the present study focused on the clinical performance of predefined EV populations rather than re-establishing EV identity.

Surface marker profiling was performed using the ExoView^®^ R100 platform (Unchained Labs, Boston, MA, USA), which enables single-particle analysis of vesicles captured on antibody-functionalized microarray spots. Captured vesicles were subsequently characterized by fluorescence-based detection of surface markers according to the manufacturer’s recommendations and previously published protocols [10].

Based on our prior polytrauma studies and biological plausibility, two predefined sEV populations were selected for further analysis: CD9⁺CD14⁺ sEVs and CD9⁺CD61⁺ sEVs. Thus, both the investigated EV populations and the primary respiratory-support endpoints were predefined before the present thoracic-trauma-focused reanalysis and were not selected based on post hoc optimization of the current dataset. CD14 is predominantly expressed by circulating monocytes and therefore CD9⁺CD14⁺ sEVs are considered monocyte-associated [10]. Thus, CD14 was chosen as a marker of monocyte-associated vesicles because monocyte activation represents a central component of the early inflammatory response following trauma. CD61 was selected to capture predominantly platelet-associated vesicles, reflecting early hemostatic and vascular responses to injury. These two populations had previously emerged as the most clinically relevant EV signals within the original cohort and were therefore prospectively carried forward into the present thoracic-trauma-focused analysis [10–12].

Absolute particle counts were used for all statistical analyses without additional normalization or transformation in order to preserve the original biological signal and facilitate comparison with previously published results from the same cohort.

### Clinical outcome parameters

The following predefined respiratory-support trajectory endpoints were analyzed:

- Tracheostomy (yes/no)
- Prolonged mechanical ventilation > 5 days (Vent > 5 d)

Prolonged ICU stay > 5 days (ICU > 5 d) was analysed as a supportive endpoint reflecting overall intensive-care burden.

Tracheostomy was performed based on interdisciplinary clinical decision-making, primarily in the context of anticipated prolonged mechanical ventilation and difficult weaning following thoracic trauma.

The following secondary sensitivity endpoints were analyzed:

- Prolonged mechanical ventilation > 10 days (Vent > 10 d)
- Prolonged ICU stay > 10 days (ICU > 10 d)

The following exploratory endpoints were evaluated:

- Early respiratory failure, defined as the requirement for mechanical ventilation within 48 h after trauma
- Respiratory failure, defined as the requirement for mechanical ventilation beyond 48 h due to impaired gas exchange
- Pneumonia, defined as a respiratory infection diagnosed by imaging, microbiological findings, bronchoscopy, or a combination thereof according to institutional standards
  Note: The term respiratory failure was used to describe the overall occurrence of ventilatory support irrespective of timing.

The predefined primary respiratory-support endpoints were tracheostomy, prolonged mechanical ventilation (> 5 days), and prolonged ICU stay (> 5 days). These endpoints were intentionally selected because they capture complementary dimensions of the same unfavourable respiratory-support trajectory rather than representing fully independent clinical entities. Together, they provide a more comprehensive characterization of respiratory-support burden following thoracic trauma than any individual endpoint alone.

Continuous outcome parameters included:

- ICU length of stay (days)
- Duration of mechanical ventilation (days)
- Clinical severity parameters included:
- Injury Severity Score (ISS)
- AIS Chest
- Thoracic Trauma Severity Score (TTSS)
- RibScore
- PaO₂/FiO₂ ratio on Day 0 and Day 1 after trauma

### Laboratory parameters

Longitudinal laboratory parameters were included to assess systemic inflammatory responses:

- White blood cell count (WBC): admission, Day 1, Day 2, Day 7, Day 10
- C-reactive protein (CRP): Day 1, Day 2, Day 7, Day 10
- Procalcitonin (PCT): Day 1, Day 2, Day 7, Day 10
- Interleukin-6 (IL-6): Day 1, Day 2, Day 7, Day 10

Additional laboratory parameters included:

- Platelet count
- Erythrocyte count
- Albumin

### Predictive models

To compare different information layers available during early trauma care, predefined approaches were evaluated. Thus, given the limited cohort size and number of outcome events, these preliminary models were intended as exploratory comparisons of predefined biomarker, clinical injury, laboratory, and oxygenation information layers rather than for the development or validation of a definitive clinical prediction model. Accordingly, the resulting AUROC estimates should be interpreted as internal comparative performance measures within the present cohort. The models are:

- CD9⁺CD14⁺ sEV Model
  - CD9⁺CD14⁺ small extracellular vesicles
- CD9⁺CD61⁺ sEV Model
  - CD9⁺CD61⁺ small extracellular vesicles
- Clinical Injury Model
  - The clinical injury model included:
  - Age
  - Injury Severity Score (ISS)
  - AIS Chest
  - RibScore
  - Pneumothorax
  - Hemothorax
  - Abdominal injury
  - Pelvic injury
  - Traumatic brain injury

### Laboratory model

The laboratory model included:

- White blood cell count at admission
- White blood cell count on Day 1
- Platelet count
- CRP on Day 1
- IL-6 on Day 1
- Albumin on Day 1

### Oxygenation-based models

PaO₂/FiO₂ ratios on Day 0 and Day 1 were evaluated as separate oxygenation-based information layers. Because traumatic brain injury (TBI) may influence early intubation and ventilatory management independent of thoracic injury severity, analyses were repeated after exclusion of patients with traumatic brain injury.

### Statistical analysis

All statistical analyses were performed using Python (version 3.11.5, Python Software Foundation, Wilmington, DE, USA) implemented in Google Colab (Google LLC, Mountain View, CA, USA). Continuous variables are presented as median with interquartile range (IQR) or mean ± standard deviation, as appropriate. Categorical variables are presented as absolute numbers and percentages. Comparisons between two independent groups were performed using the Mann–Whitney U test. Categorical variables were analyzed using Fisher’s exact test or χ² test, as appropriate. Correlations between EV subpopulations, clinical parameters, and laboratory markers were assessed using Spearman’s rank correlation coefficient (r_SP_). Correlation matrices were visualized as heat maps including significance annotations (*p* < 0.05, *p* < 0.01, and *p* < 0.001). The discriminatory performance of EV-associated biomarkers, clinical injury variables, laboratory parameters, and oxygenation-based approaches was evaluated using supervised machine-learning models. Logistic regression models were implemented within a standardized pipeline incorporating median imputation and feature scaling. Model performance was assessed using repeated stratified three-fold cross-validation with 30 repeats. Receiver operating characteristic (ROC) curves were generated from predicted probabilities, and the area under the ROC curve (AUROC) was calculated as a measure of classification performance. AUROC values are reported as mean ± standard deviation across cross-validation iterations. Differences in predictive performance across competing models were first assessed using the Friedman test as a global non-parametric comparison. When significant, pairwise comparisons were performed using Wilcoxon signed-rank tests with Holm correction for multiple testing. In an exploratory analysis, residual CD9⁺CD14⁺ signals were calculated to investigate whether EV-associated information extended beyond established measures of thoracic injury severity. Expected CD9⁺CD14⁺ values were estimated using separate linear regression models based on AIS Chest and TTSS. Residual values were calculated as the difference between observed and predicted CD9⁺CD14⁺ levels. Patients were subsequently classified as having higher-than-expected or expected/lower-than-expected CD9⁺CD14⁺ levels relative to injury severity. Associations with respiratory-support trajectory endpoints were evaluated using contingency tables, Fisher’s exact test, and odds ratios. Missing data were handled using median imputation within the machine-learning pipeline. All statistical tests were two-sided, and a p-value < 0.05 was considered statistically significant.

## Results

### Patient cohort description

A total of 45 adult patients with confirmed thoracic trauma were included in this analysis. The mean age of the cohort was 53.6 ± 16.9 years (range 20–79), and the majority of patients were male (77.8%, 35/45). The mean body mass index (BMI) was 27.3 ± 3.8 kg/m². The overall injury burden reflected a severely affected trauma population, with a mean Injury Severity Score (ISS) of 30.7 ± 12.4. Thoracic injury severity was moderate to severe, with a mean Thoracic Trauma Severity Score (TTSS) of 6.8 ± 3.6, an AIS chest of 2.9 ± 1.2, and a RibScore of 1.9 ± 1.4.

Fourteen patients (31.1%) underwent tracheostomy during their clinical course, whereas 31 patients (68.9%) did not. Patients requiring tracheostomy exhibited significantly higher injury severity compared to those without tracheostomy, as reflected by higher ISS (39.6 ± 13.1 vs. 26.7 ± 9.9, *p* = 0.005), TTSS (9.0 ± 3.9 vs. 5.7 ± 2.9, *p* = 0.010), and AIS chest (3.6 ± 1.1 vs. 2.6 ± 1.1, *p* = 0.008). RibScore did not differ significantly between groups.

Functional outcomes differed markedly between groups. Patients undergoing tracheostomy required significantly longer mechanical ventilation (23.1 ± 13.6 vs. 1.1 ± 2.7 days, *p* < 0.001) and prolonged ICU stay (26.4 ± 13.9 vs. 3.6 ± 3.9 days, *p* < 0.001).

Respiratory complications were more frequent in the tracheostomy group, including respiratory failure requiring mechanical ventilation (92.9% vs. 19.4%, *p* < 0.001), early respiratory failure (78.6% vs. 22.6%, *p* < 0.001), and pneumonia (42.9% vs. 9.7%, *p* = 0.017). Pneumothorax was more frequently observed in patients requiring tracheostomy (64.3% vs. 25.8%, *p* = 0.021), whereas no clear differences were detected for other injury patterns, including hemothorax, abdominal trauma, and pelvic injury. Pulmonary contusions were numerically more frequent among patients requiring tracheostomy, although this association did not reach statistical significance in the present cohort. Importantly, in the present cohort TBI was not more frequent in patients requiring tracheostomy (21% vs. 32%), and severe head injury (AIS head > 3) showed a comparable distribution between groups.

These findings suggest that thoracic injury and respiratory compromise contributed substantially to tracheostomy requirement in this cohort. However, the influence of neurological status, overall trauma burden, and other clinical factors cannot be fully excluded.

Baseline laboratory parameters, including WBC, CRP, IL-6, and albumin at day 1, did not differ significantly between groups. Longitudinal laboratory measurements demonstrated a typical inflammatory response pattern following trauma (Table 1).

Delayed respiratory failure, defined as need for intubation after 48 h on ICU occurred in only two patients (4.4%) and was therefore not included in further statistical analyses due to insufficient sample size. All patients had serum samples collected within 24 h after trauma admission for subsequent sEV profiling.

### Extracellular vesicle levels by clinical outcome

**Fig. 1:**
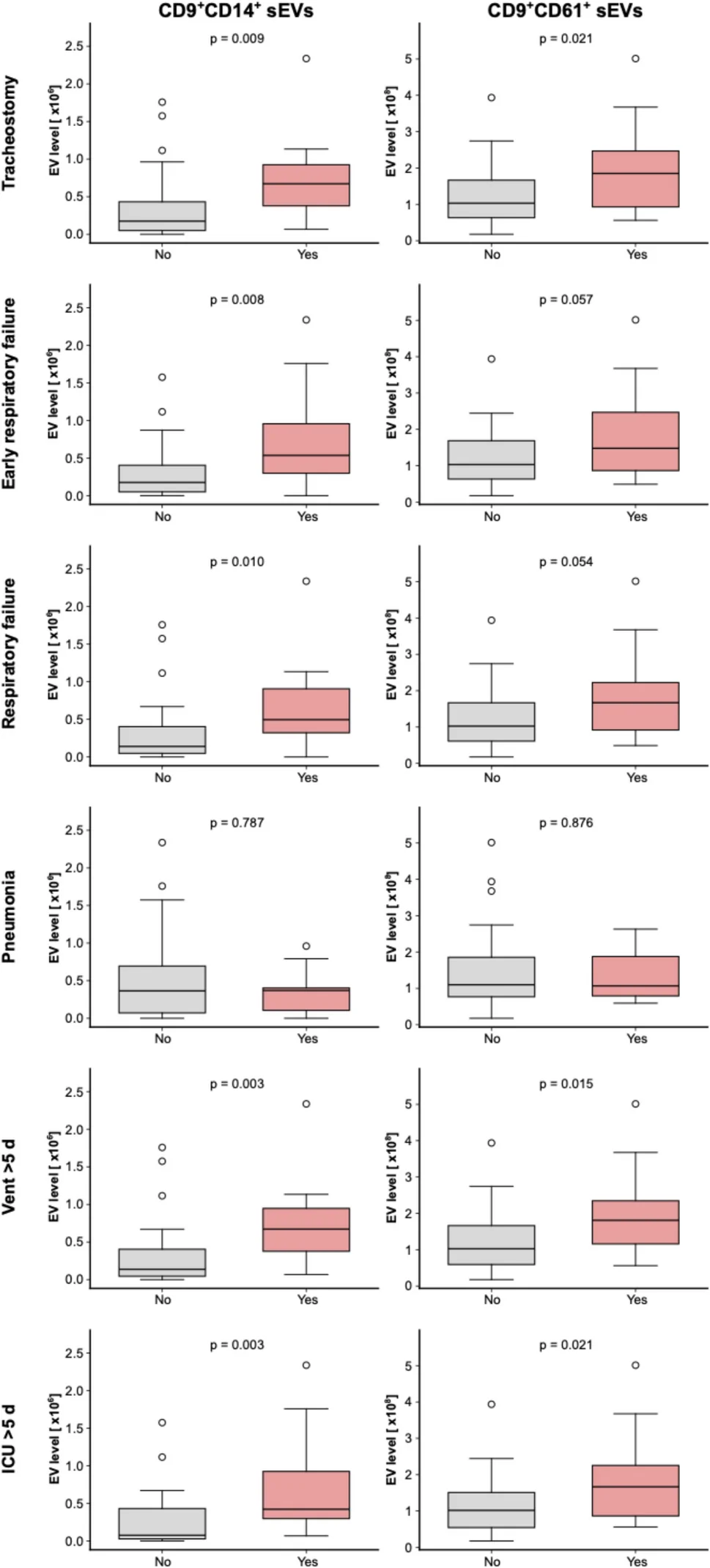
Extracellular vesicle levels according to clinical outcomes in thoracic trauma. Boxplots display circulating levels of CD9⁺CD14⁺ and CD9⁺CD61⁺ small extracellular vesicles (sEVs) stratified by clinically relevant outcome parameters, including tracheostomy, early respiratory failure, overall respiratory failure, pneumonia, prolonged mechanical ventilation (> 5 days), and prolonged ICU stay (> 5 days). For each endpoint, patients are grouped into those without (“No”) and with (“Yes”) the respective outcome. Boxes represent the interquartile range (IQR) with the median indicated by a horizontal line; whiskers indicate 1.5 × the IQR, and outliers are shown as individual points. Statistical comparisons between groups were performed using the two-sided Mann–Whitney U test, with corresponding p-values indicated within each panel. CD9⁺CD14⁺ sEV levels are generally higher in patients with adverse clinical outcomes, whereas CD9⁺CD61⁺ sEVs show a similar but less pronounced pattern. No significant differences were observed for pneumonia

Levels of CD9⁺CD14⁺ and CD9⁺CD61⁺ sEVs were assessed across clinically relevant outcome parameters in patients with thoracic trauma (Fig. 1). A consistent pattern emerged for CD9⁺CD14⁺ sEVs, which were elevated in patients with more severe clinical courses. CD9⁺CD14⁺ sEV levels were markedly higher in patients requiring tracheostomy compared to those without (median 671,500 vs. 175,000, *p* = 0.009), corresponding to a 3.84-fold increase. CD9⁺CD61⁺ sEVs showed a similar but less pronounced increase (median 185,176,500 vs. 103,156,900, *p* = 0.021; 1.80-fold increase). A comparable pattern was observed for early respiratory failure. CD9⁺CD14⁺ sEV levels were substantially elevated in affected patients (median 535,500 vs. 175,000, *p* = 0.008), representing a 3.06-fold increase. CD9⁺CD61⁺ sEV levels were also higher in this subgroup, although the difference did not reach statistical significance (median 147,841,600 vs. 103,156,900, *p* = 0.057; 1.43-fold increase). This trend remained evident when considering respiratory failure more broadly. CD9⁺CD14⁺ sEV levels clearly distinguished patients with respiratory compromise (median 496,000 vs. 140,000, *p* = 0.010), corresponding to a 3.54-fold increase. In contrast, CD9⁺CD61⁺ sEV levels showed only a moderate, non-significant increase (median 167,000,000 vs. 102,505,950, *p* = 0.054; 1.63-fold increase). In contrast to these findings, no meaningful differences were observed in relation to pneumonia. CD9⁺CD14⁺ sEV levels were comparable between patients with and without pneumonia (median 370,000 vs. 364,474, *p* = 0.787), and a similar absence of separation was observed for CD9⁺CD61⁺ sEVs (median 106,885,510 vs. 110,027,750, *p* = 0.876). The association between elevated sEV levels and more severe clinical trajectories became particularly evident when examining short-term organ support. CD9⁺CD14⁺ sEV levels were markedly elevated in patients requiring mechanical ventilation for more than 5 days (median 671,500 vs. 137,500, *p* = 0.003), corresponding to a 4.88-fold increase. CD9⁺CD61⁺ sEVs were also significantly higher in this group (median 180,914,000 vs. 102,622,500, *p* = 0.015; 1.76-fold increase).

A similar pattern was observed for ICU stay exceeding 5 days (Fig. 1). CD9⁺CD14⁺ sEV levels were higher in patients with prolonged ICU stay (median 421,725 vs. 75,000, *p* = 0.003), corresponding to a 5.62-fold increase. CD9⁺CD61⁺ sEV levels were likewise increased (median 166,638,500 vs. 101,855,000, *p* = 0.021; 1.64-fold increase).

### Relationships between EVs, injury severity, and respiratory support trajectories

**Fig. 2:**
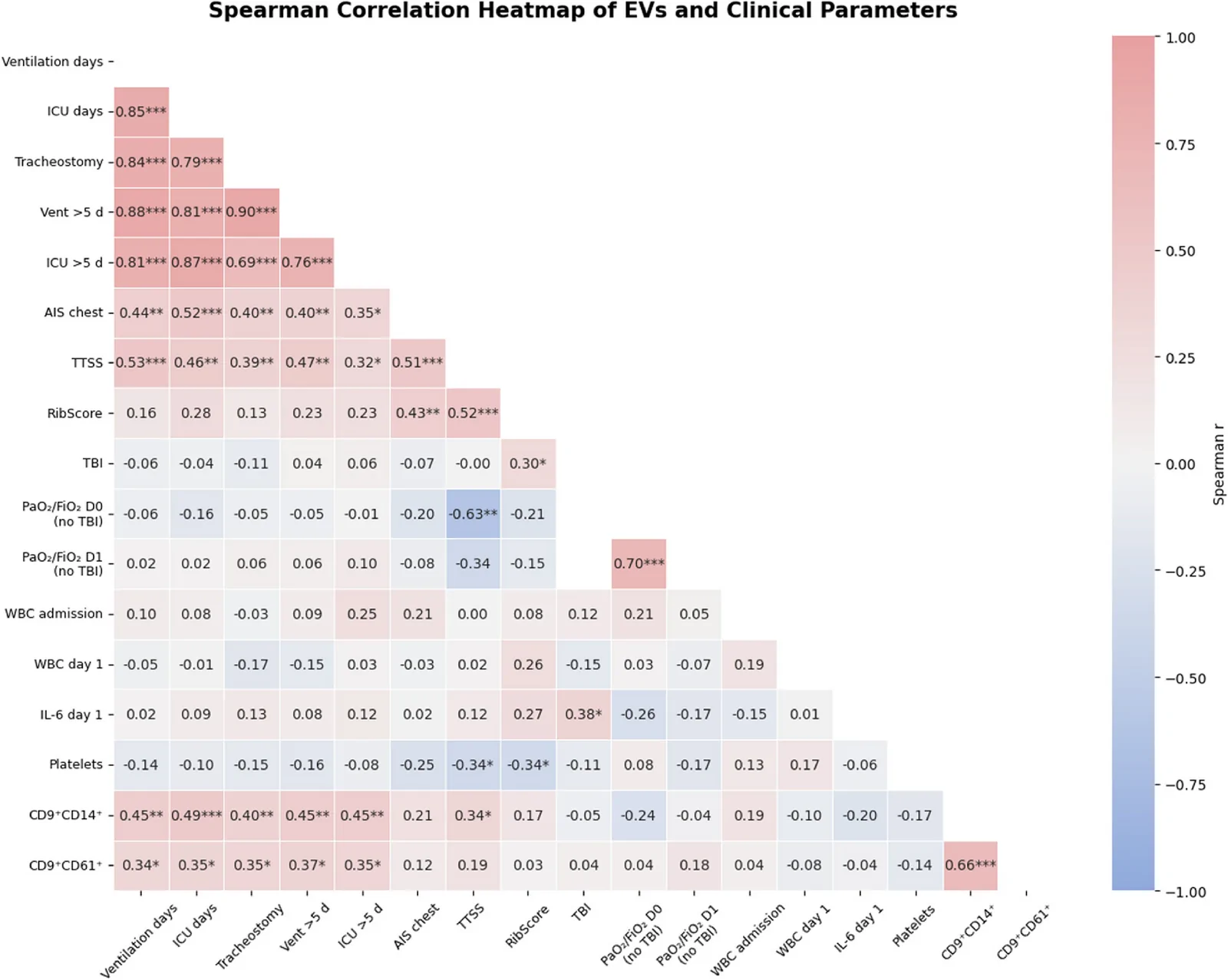
Spearman correlation heatmap of extracellular vesicles, injury severity parameters, oxygenation indices, laboratory markers, and respiratory support trajectories in patients with thoracic trauma. Pairwise Spearman rank correlation coefficients (r_Sp_) are shown for clinical respiratory-support endpoints, thoracic injury severity scores, oxygenation indices, laboratory parameters, and extracellular vesicle (EV) populations. Positive correlations are displayed in red and negative correlations in blue, with color intensity corresponding to correlation strength. Only the lower triangular matrix is displayed. Clinical respiratory-support endpoints included ventilation duration, ICU length of stay, tracheostomy, prolonged ventilation (> 5 days), and prolonged ICU stay (> 5 days). Injury severity parameters comprised AIS Chest, Thoracic Trauma Severity Score (TTSS), RibScore, and traumatic brain injury (TBI). Oxygenation was assessed using PaO₂/FiO₂ ratios on Day 0 and Day 1 after exclusion of patients with traumatic brain injury to reduce bias introduced by pre-hospital or early mechanical ventilation. Laboratory parameters included admission leukocyte count, leukocyte count on Day 1, IL-6 on Day 1, and platelet count. EV populations were represented by CD9⁺CD14⁺ and CD9⁺CD61⁺ small extracellular vesicles. Correlation coefficients are displayed within each cell. Statistical significance is indicated as follows: *p < 0.05, **p < 0.01, ***p < 0.001

Spearman correlation (r_Sp_) analysis demonstrated strong associations among the clinically relevant respiratory support endpoints (Fig. 2). Ventilation duration correlated strongly with ICU stay (r_Sp_ = 0.85, *p* < 0.001), tracheostomy (r_Sp_ = 0.84, *p* < 0.001), and prolonged ventilation > 5 days (r_Sp_ = 0.88, *p* < 0.001).

Thoracic injury severity scores were moderately associated with respiratory support requirements. AIS Chest correlated with ventilation duration (r_Sp_ = 0.44, *p* < 0.01), ICU stay (r_Sp_ = 0.52, *p* < 0.001), tracheostomy (r_Sp_ = 0.40, *p* < 0.01), and prolonged ventilation > 5 days (r_Sp_ = 0.40, *p* < 0.01). Similar associations were observed for TTSS, which correlated with ventilation duration (r_Sp_ = 0.53, *p* < 0.001), ICU stay (r_Sp_ = 0.46, *p* < 0.01), tracheostomy (r_Sp_ = 0.39, *p* < 0.01), and prolonged ventilation > 5 days (r_Sp_ = 0.47, *p* < 0.001).

CD9⁺CD14⁺ small extracellular vesicles demonstrated moderate positive correlations with ventilation duration (r_Sp_ = 0.45, *p* < 0.01), ICU stay (r_Sp_ = 0.49, *p* < 0.001), tracheostomy (r_Sp_ = 0.40, *p* < 0.01), prolonged ventilation > 5 days (r_Sp_ = 0.45, *p* < 0.01), and ICU stay > 5 days (r_Sp_ = 0.45, *p* < 0.01). Weaker but significant correlations were observed between CD9⁺CD61⁺ sEVs and the same endpoints (r_Sp_ = 0.34–0.37, all *p* < 0.05).

PaO₂/FiO₂ ratios at Day 0 and Day 1 after exclusion of patients with traumatic brain injury showed only weak associations with respiratory support endpoints. No meaningful correlations were observed between routine inflammatory laboratory parameters and prolonged respiratory support trajectories. A moderate positive correlation was observed between CD9⁺CD14⁺ and CD9⁺CD61⁺ sEV populations (r_Sp_ = 0.66, *p* < 0.001).

Overall, CD9⁺CD14⁺ sEVs were consistently associated with unfavorable respiratory-support trajectories following thoracic trauma, including prolonged mechanical ventilation and tracheostomy.

### Predictive performance of early information layers for respiratory-support trajectories

**Fig. 3:**
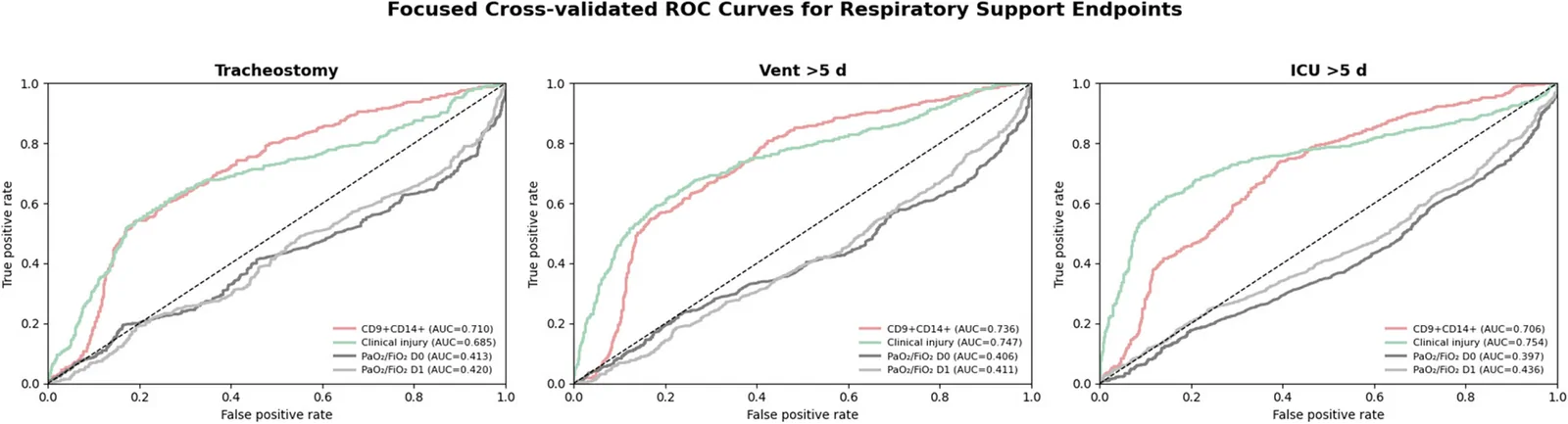
Predictive performance of early information layers for predefined respiratory-support trajectories after thoracic trauma. Cross-validated receiver operating characteristic (ROC) curves comparing the predictive performance of six early information layers available after thoracic trauma: CD9⁺CD14⁺ small extracellular vesicles (sEVs), CD9⁺CD61⁺ sEVs, a clinical injury model (age, ISS, AIS chest, RibScore, pneumothorax, hemothorax, abdominal injury, pelvic injury, and traumatic brain injury), a laboratory model (white blood cell count, platelets, CRP, IL-6, and albumin), and PaO₂/FiO₂ ratios on Day 0 and Day 1 after exclusion of traumatic brain injury cases. Primary respiratory-support endpoints included tracheostomy and prolonged mechanical ventilation (> 5 days). Prolonged ICU stay (> 5 days) was analyzed as a supportive endpoint reflecting overall intensive-care burden. Mean AUROC values were derived from repeated stratified cross-validation (3-fold, 30 repeats). The diagonal dashed line represents random classification performance (AUROC = 0.5). For clarity, the main figure displays the focused comparison between CD9⁺CD14⁺ sEVs, the clinical injury model, and PaO₂/FiO₂-based models. Full model comparisons including CD9⁺CD61⁺ sEVs and the laboratory model are provided in Supplementary Figure S1

Having established associations between EV levels and respiratory-support trajectories, we next evaluated whether EV-associated information could help identify patients who subsequently required prolonged respiratory support. To place EV performance into a clinical context, predictive models based on EV markers, anatomical injury severity, routine laboratory parameters, and oxygenation indices were compared using repeated cross-validated AUROC analysis (Fig. 3; Table 2).

**Table 2:** Cross-validated predictive performance of early information layers for respiratory-support trajectories after thoracic trauma

endpoint	comparison	p_raw	p_holm	p_raw_formatted	p_holm_formatted
Tracheostomy	CD9^+^CD14^+^ vs. CD9^+^CD61^+^	2.340586 × 10^− 2^	1.404352 × 10^− 1^	0.023	0.140
Tracheostomy	CD9^+^CD14^+^ vs. Clinical injury	4.250024 × 10^− 2^	2.125012 × 10^− 1^	0.043	0.213
Tracheostomy	CD9^+^CD14^+^ vs. Laboratory model	2.633747 × 10–^16^	3.687246 × 10^− 15^	< 0.001	< 0.001
Vent > 5 d	CD9^+^CD14^+^ vs. CD9 + CD61+	2.333642 × 10^− 3^	1.400185 × 10^− 2^	0.002	0.014
Vent > 5 d	CD9^+^CD14^+^ vs. Clinical injury	5.379904 × 10^− 1^	5.379904 × 10^− 1^	0.538	0.538
Vent > 5 d	CD9^+^CD14^+^ vs. Laboratory model	6.339882 × 10^− 16^	5.978375 × 10^− 15^	< 0.001	< 0.001
ICU > 5 d	CD9^+^CD14^+^ vs. CD9 + CD61+	1.058785 × 10^− 6^	5.293924 × 10^− 6^	< 0.001	< 0.001
ICU > 5 d	CD9^+^CD14^+^ vs. Clinical injury	6.158620 × 10^− 1^	6.158620 × 10^− 1^	0.616	0.616
ICU > 5 d	CD9^+^CD14^+^ vs. Laboratory model	7.188155 × 10^− 12^	5.750524 × 10^− 11^	< 0.001	< 0.001

Mean AUROC ± standard deviation obtained from repeated stratified cross-validation (3-fold, 30 repeats) for EV-associated biomarkers, clinical injury variables, routine laboratory parameters, and PaO₂/FiO₂-based models. The clinical injury model comprised age, ISS, AIS chest, RibScore, pneumothorax, hemothorax, abdominal injury, pelvic injury, and traumatic brain injury. The laboratory model comprised white blood cell count, platelets, CRP, IL-6, and albumin. PaO₂/FiO₂ Day 0 and Day 1 analyses were performed after exclusion of patients with traumatic brain injury to reduce bias from early intubation and ventilation-related interventions. Primary respiratory-support endpoints were tracheostomy and prolonged mechanical ventilation (> 5 days). Prolonged ICU stay (> 5 days) was retained as a supportive endpoint reflecting overall intensive-care burden. Respiratory failure, early respiratory failure, pneumonia, and severe trajectory endpoints (> 10 days) were analyzed as exploratory or sensitivity endpoints. Respiratory failure, early respiratory failure, pneumonia, and severe trajectory endpoints (> 10 days) were analyzed as exploratory or sensitivity endpoints. Global differences between information layers were assessed using the Friedman test. Pairwise comparisons were performed using Wilcoxon signed-rank tests with Holm correction for multiple testing

For tracheostomy prediction, CD9⁺CD14⁺ sEVs achieved a mean AUROC of 0.743 ± 0.100, compared with 0.702 ± 0.134 for the clinical injury model, 0.716 ± 0.123 for CD9⁺CD61⁺ sEVs, 0.417 ± 0.143 for the laboratory model, and 0.413 ± 0.141 and 0.431 ± 0.150 for PaO₂/FiO₂ Day 0 and Day 1, respectively (Table 2) under the conditions of this exploratory cohort.

For prolonged ventilation (> 5 days), CD9⁺CD14⁺ sEVs yielded a mean AUROC of 0.771 ± 0.104. Comparable performance was observed for the clinical injury model (0.766 ± 0.119), whereas lower performance was observed for CD9⁺CD61⁺ sEVs (0.727 ± 0.111), the laboratory model (0.470 ± 0.151), and PaO₂/FiO₂-based models (0.415 ± 0.131 and 0.432 ± 0.121 for Day 0 and Day 1, respectively).

For prolonged ICU stay (> 5 days), CD9⁺CD14⁺ sEVs achieved a mean AUROC of 0.757 ± 0.098, which was similar to the clinical injury model (0.766 ± 0.112). Lower predictive performance was observed for CD9⁺CD61⁺ sEVs (0.702 ± 0.112), the laboratory model (0.601 ± 0.122), and PaO₂/FiO₂-based models (0.408 ± 0.097 and 0.463 ± 0.133 for Day 0 and Day 1, respectively).

In contrast, predictive performance for pneumonia differed from the respiratory-support endpoints. The highest AUROC was observed for PaO₂/FiO₂ Day 0 (0.624 ± 0.163), whereas EV-based models, the clinical injury model, and the laboratory model showed lower predictive performance (Table 2).

Global model comparison using the Friedman test demonstrated significant differences among information layers across all evaluated endpoints (all *p* < 0.001). Consistent with the exploratory design of the present study, pairwise Holm-corrected comparisons for the predefined primary respiratory-support endpoints showed no significant difference between CD9⁺CD14⁺ sEVs and the clinical injury model for tracheostomy (*p* = 0.213), prolonged ventilation > 5 days (*p* = 0.538), or ICU stay > 5 days (*p* = 0.616). Within this exploratory cohort, CD9⁺CD14⁺ sEVs demonstrated significantly higher discriminatory performance than the laboratory model across all three endpoints (all *p* < 0.001). Compared with CD9⁺CD61⁺ sEVs, CD9⁺CD14⁺ demonstrated higher discriminatory performance for prolonged ventilation > 5 days (*p* = 0.014) and ICU stay > 5 days (*p* < 0.001), whereas no significant difference was observed for tracheostomy (*p* = 0.140).

The predefined primary respiratory-support endpoints were tracheostomy and prolonged mechanical ventilation (> 5 days), whereas ICU stay > 5 days was retained as a supportive endpoint reflecting overall intensive-care burden.

Similar findings were observed in sensitivity analyses applying a more stringent definition of prolonged respiratory-support trajectories (> 10 days). For prolonged mechanical ventilation exceeding 10 days, CD9⁺CD14⁺ sEVs achieved the highest predictive performance (AUROC 0.808 ± 0.115), exceeding both the clinical injury model (AUROC 0.741 ± 0.111) and CD9⁺CD61⁺ sEVs (AUROC 0.726 ± 0.132). In contrast, for prolonged ICU stay exceeding 10 days, the clinical injury model demonstrated the strongest predictive performance (AUROC 0.834 ± 0.094), followed by CD9⁺CD14⁺ sEVs (AUROC 0.745 ± 0.107). Overall, the pattern observed for the predefined > 5-day endpoints remained largely unchanged under the more stringent > 10-day threshold. Detailed results are provided in Supplementary Figure S2 and Supplementary Tables S2–S2b.

Although predictive performance of CD9⁺CD14⁺ sEVs was comparable to that of the clinical injury model, these analyses do not clarify whether both approaches capture the same underlying information. We therefore next explored whether the EV signal merely reflects anatomical injury severity or whether it provides additional biological information beyond established thoracic injury scores.

### Residual CD9⁺CD14⁺ signal beyond anatomical injury severity

**Fig. 4:**
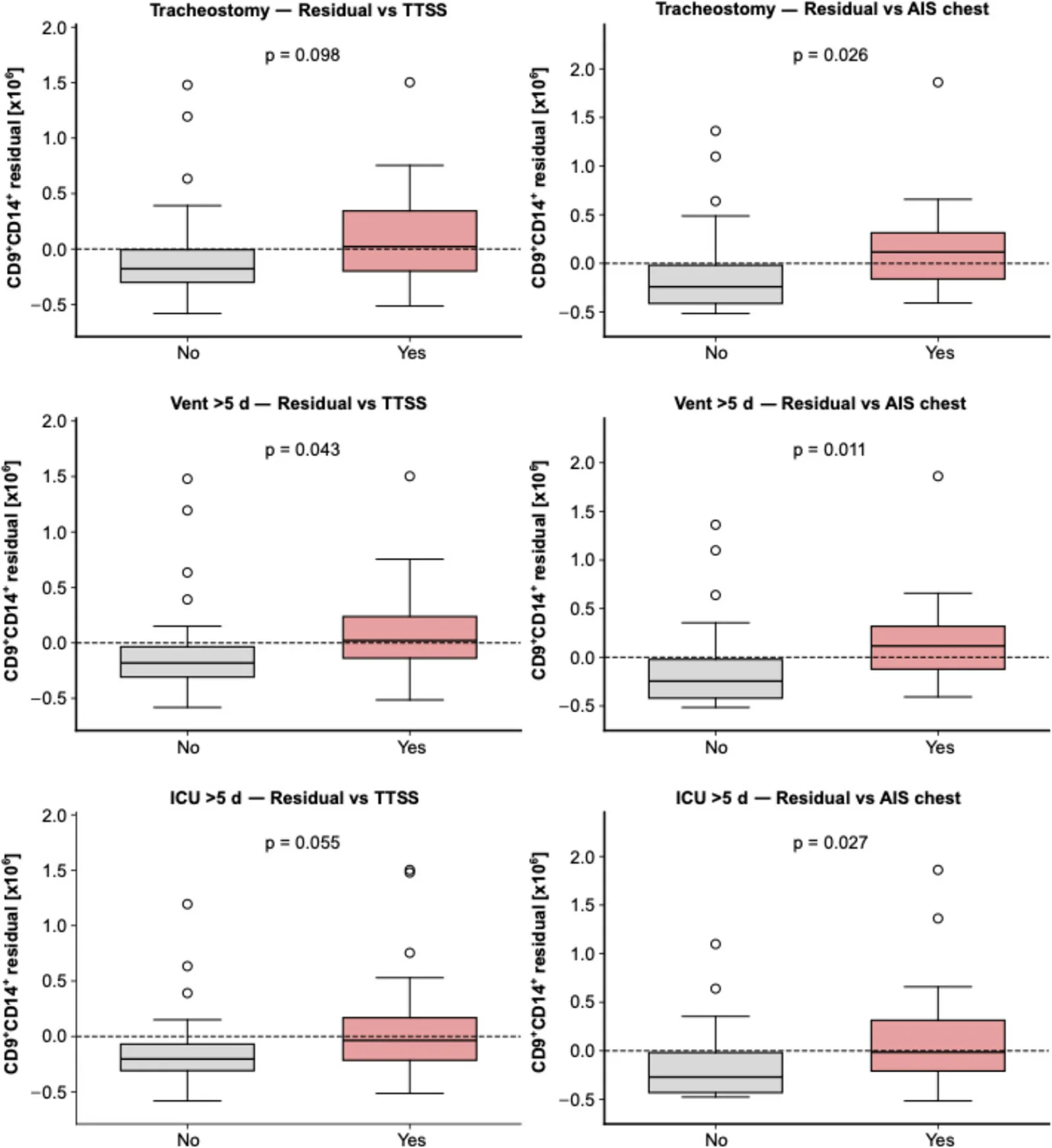
Residual CD9⁺CD14⁺ signal relative to thoracic injury severity and association with respiratory-support trajectories. Expected CD9⁺CD14⁺ small extracellular vesicle (sEV) levels were estimated from thoracic injury severity using separate linear regression models based on the Thoracic Trauma Severity Score (TTSS) and AIS Chest. Residual CD9⁺CD14⁺ values were calculated as the difference between observed and expected EV levels. Patients were subsequently classified as having either higher-than-expected or expected/lower-than-expected CD9⁺CD14⁺ levels relative to their injury severity. Panels depict the distribution of residual CD9⁺CD14⁺ values according to the predefined respiratory-support trajectory endpoints of tracheostomy, prolonged mechanical ventilation (> 5 days), and prolonged ICU stay (> 5 days). Dashed lines indicate the expected EV level predicted from injury severity. Patients with positive residuals exhibit higher CD9⁺CD14⁺ levels than expected for the degree of thoracic injury, whereas patients with negative residuals exhibit lower levels than expected. This exploratory analysis was performed to investigate whether CD9⁺CD14⁺ sEVs provide information beyond established measures of anatomical thoracic injury severity

To explore whether the CD9⁺CD14⁺ sEV signal merely reflected thoracic injury severity or provided additional information, expected CD9⁺CD14⁺ levels were estimated from TTSS and AIS Chest using linear regression models. Residual CD9⁺CD14⁺ values were calculated as the difference between observed and expected EV levels. Patients were subsequently classified as having either higher-than-expected or expected/lower-than-expected CD9⁺CD14⁺ levels relative to their injury severity (Fig. 4; Table 3).

**Table 3:** Association between residual CD9⁺CD14⁺ signal and respiratory-support trajectories after adjustment for thoracic injury severity

Residual signal	Endpoint	Expected/lower EV: no endpoint	Expected/lower EV: endpoint	Expected/lower EV: endpoint rate (%)	Higher-than-expected EV: no endpoint	Higher-than-expected EV: endpoint	Higher-than-expected EV: endpoint rate (%)	Odds ratio	Fisher *p*	Fisher *p* formatted
Residual vs. TTSS	Tracheostomy	23	7	23.3	8	7	46.7	2.875	0.172266	0.172
Residual vs. TTSS	Vent > 5 d	22	8	26.7	7	8	53.3	3.142857	0.104519	0.105
Residual vs. TTSS	ICU > 5 d	18	12	40.0	5	10	66.7	3.0	0.120451	0.120
Residual vs. AIS chest	Tracheostomy	24	5	17.2	7	9	56.2	6.171429	0.016353	0.016
Residual vs. AIS chest	Vent > 5 d	23	6	20.7	6	10	62.5	6.388889	0.008779	0.009
Residual vs. AIS chest	ICU > 5 d	18	11	37.9	5	11	68.8	3.6	0.065387	0.065

Patients were classified according to whether observed CD9⁺CD14⁺ small extracellular vesicle (sEV) levels were higher than expected or expected/lower than expected based on linear regression models using TTSS or AIS Chest. Contingency analyses were then performed for the predefined respiratory-support trajectory endpoints of tracheostomy, prolonged mechanical ventilation (> 5 days), and prolonged ICU stay (> 5 days). For each endpoint, the table reports the number of patients with and without the endpoint, endpoint frequencies, odds ratios, and Fisher’s exact test p-values. Odds ratios greater than 1 indicate an increased frequency of unfavorable respiratory-support trajectories among patients with higher-than-expected CD9⁺CD14⁺ levels relative to their anatomical thoracic injury severity

Using TTSS-adjusted residuals, patients with higher-than-expected CD9⁺CD14⁺ levels showed numerically increased frequencies of tracheostomy (Higher-than-expected EV: endpoint rate: 46.7% vs. 23.3%), prolonged mechanical ventilation > 5 days (Higher-than-expected EV: endpoint rate: 53.3% vs. 26.7%), and prolonged ICU stay > 5 days ( Higher-than-expected EV: endpoint rate: 66.7% vs. 40.0%) compared with patients whose CD9⁺CD14⁺ levels were at or below those predicted by TTSS (Table 3). Corresponding odds ratios (Table 3) ranged from 2.88 to 3.14; however, these associations did not reach statistical significance (all *p* > 0.05).

In contrast, AIS Chest-adjusted residual analyses demonstrated stronger associations. Patients with higher-than-expected CD9⁺CD14⁺ levels exhibited increased rates of tracheostomy (56.2% vs. 17.2%; OR 6.17, *p* = 0.016) and prolonged mechanical ventilation > 5 days (62.5% vs. 20.7%; OR 6.39, *p* = 0.009) compared with patients whose CD9⁺CD14⁺ levels were consistent with or below those predicted by AIS Chest. A similar pattern was observed for prolonged ICU stay > 5 days (68.8% vs. 37.9%; OR 3.60), although statistical significance was not reached (*p* = 0.065).

Overall, patients with higher-than-expected CD9⁺CD14⁺ levels relative to anatomical thoracic injury severity experienced less favorable respiratory-support trajectories than patients with EV levels consistent with the degree of thoracic injury.

Taken together, these exploratory analyses suggest that early CD9⁺CD14⁺ sEV levels were consistently associated with predefined respiratory-support trajectories following thoracic trauma. While anatomical injury severity remained an important determinant of outcome, elevated CD9⁺CD14⁺ levels relative to the degree of thoracic injury identified patients with an increased likelihood of tracheostomy, prolonged mechanical ventilation, and prolonged ICU treatment. These findings suggest that EV-associated information and established injury severity measures may capture complementary aspects of the post-traumatic response.

## Discussion

Trauma scoring systems were developed to predict mortality and guide triage [22]; however, their performance remains inconsistent across patient populations. Anatomical models such as the Injury Severity Score (ISS) and New Injury Severity Score (NISS) summarize injury burden based on Abbreviated Injury Scale (AIS) values [23, 24], while the Trauma and Injury Severity Score (TRISS) integrates anatomical and physiological parameters [25]. While these models support population-level risk assessment, their value in predicting patient-specific outcomes or guiding procedural decisions remains less clear. Notably, no universally accepted clinical prediction model exists for blunt chest trauma [3]. The Thoracic Trauma Severity Score (TTSS) introduced a more comprehensive framework incorporating age, gas exchange, and radiological findings [4], but lacks defined cut-offs for ICU admission or mechanical ventilation and may underestimate injuries detectable only by CT imaging [26].

Against this background, our exploratory pilot study investigated whether early small extracellular vesicle (sEV) measurements may identify patients who subsequently follow unfavorable respiratory-support trajectories after thoracic trauma. Rather than focusing on a single physiological endpoint, we evaluated clinically relevant outcomes reflecting respiratory-support requirements, including tracheostomy, prolonged mechanical ventilation, and prolonged ICU treatment. Hence, these endpoints should be interpreted as complementary manifestations of the same respiratory-support trajectory rather than as independent biological outcomes.

In our proof-of-concept exploratory cohort, patients requiring tracheostomy represented a clinically more severe subgroup characterized by higher injury severity scores, increased rates of respiratory failure, and substantially prolonged ICU and ventilation duration. These findings confirm that the investigated endpoints reflect meaningful gradients of injury severity and resource utilization, providing a clinically relevant framework for biomarker evaluation. Although tracheostomy is a clinically relevant endpoint reflecting prolonged respiratory-support requirements, its occurrence may also be influenced by factors beyond thoracic injury itself, including neurological status, airway protection, secretion burden, sedation requirements, local weaning protocols, and center-specific clinical practice patterns [27]. In our exploratory cohort, tracheostomy was predominantly associated with prolonged ventilatory dependence following thoracic trauma. Nevertheless, contributions from neurological status, overall injury burden, and local clinical practice patterns cannot be excluded.

The most consistent experimental biomarker signal observed in this study was provided by CD9⁺CD14⁺ monocyte-associated sEVs. Elevated levels were associated with tracheostomy, prolonged mechanical ventilation, prolonged ICU treatment, respiratory failure, and early respiratory failure. But we have to acknowledge that even tracheostomy represents a clinically meaningful endpoint, the decision to perform tracheostomy is influenced by multiple factors beyond thoracic injury itself, including institutional practice, sedation strategy, neurological status, airway protection, and weaning success. Accordingly, the observed association between CD9⁺CD14⁺ sEVs and tracheostomy should be interpreted as reflecting the broader respiratory-support trajectory.

In contrast, CD9⁺CD61⁺ sEVs (mainly platelet associated) demonstrated similar but generally weaker associations. Importantly, neither EV population was associated with pneumonia in the present cohort. While this finding should be interpreted cautiously given the limited cohort size, it suggests that the observed EV signal cannot be explained solely by the occurrence of subsequent pulmonary infection.

A notable observation was the performance hierarchy across the investigated information layers. This raises an important question: do our investigated EV population simply mirror established clinical information, or do they capture a distinct biological signal? The clinical injury model, incorporating age, ISS, AIS Chest, RibScore, pneumothorax, hemothorax, abdominal injury, pelvic injury, and traumatic brain injury, together with the monocyte-associated CD9⁺CD14⁺ sEV population, consistently demonstrated the strongest predictive performance within this exploratory for respiratory-support trajectories. In contrast, the laboratory model, comprising admission and day 1 leukocyte counts, platelet count, CRP day 1, IL-6 day 1, and albumin day 1, as well as PaO₂/FiO₂-based approaches, showed substantially lower predictive capacity. The poor performance of the laboratory model is particularly noteworthy, as it suggests that the EV signal is not merely a surrogate of routine inflammatory markers. Instead, the investigated EV populations appear to represent a distinct biological information layer.

The findings also raise the question of why oxygenation-based markers performed less well than expected despite their established role in thoracic trauma and critical care. The PaO₂/FiO₂ ratio is widely used in critical care as a measure of gas exchange impairment and forms the basis of established definitions of acute respiratory distress syndrome [28, 29]. However, interpretation of early PaO₂/FiO₂ values in trauma remains complex. The ratio is influenced not only by pulmonary injury itself but also by ventilatory settings, positive end-expiratory pressure, fraction of inspired oxygen, timing of measurement, sedation, recruitment maneuvers, and prehospital airway management [30–33]. Consequently, while PaO₂/FiO₂ reflects established physiological impairment, its dependence on ventilatory conditions and timing of assessment may limit its ability to reflect the upstream biological processes as an early surrogate for the upstream biological processes that contribute to later respiratory-support requirements. Importantly, all PaO₂/FiO₂ measurements in the present cohort were obtained during routine clinical care and therefore reflect the heterogeneity of real-world trauma management. Early oxygenation values may have been influenced by prehospital and in-hospital interventions, including endotracheal intubation, sedation, mechanical ventilation, oxygen supplementation, and recruitment strategies. This is particularly relevant in patients with traumatic brain injury, who were frequently intubated primarily for neurological indications rather than respiratory compromise. To address this potential confounding effect, additional analyses were performed using Day 0 and Day 1 PaO₂/FiO₂ models after exclusion of patients with traumatic brain injury. Despite this adjustment, experimental oxygenation-based models demonstrated lower discriminatory performance for subsequent respiratory-support trajectories than CD9⁺CD14⁺ sEVs and the clinical injury model within the present exploratory cohort. However, these findings should not be interpreted as indicating that EVs are inherently superior to established oxygenation indices. Rather, they support the hypothesis that early CD9⁺CD14⁺ sEV profiling may capture complementary biological information that is not fully reflected by routine oxygenation measurements obtained under heterogeneous real-world trauma care conditions.

The sensitivity analyses using stricter thresholds for prolonged organ support (> 10 days) further reinforced the distinction between anatomical injury burden and biological response. For prolonged mechanical ventilation exceeding 10 days, CD9⁺CD14⁺ sEVs achieved the highest predictive performance among the investigated biomarkers and performed significantly better than both CD9⁺CD61⁺ sEVs and the clinical injury model. In contrast, prolonged ICU stay exceeding 10 days showed the highest predictive performance in the clinical injury model. This divergence may indicate that prolonged ventilatory dependence is more closely linked to biological processes occurring after thoracic trauma, whereas prolonged ICU treatment reflects the overall burden of injury and associated complications.

If CD9⁺CD14⁺ sEVs merely reflected thoracic injury severity, little additional information would be expected after adjustment for AIS Chest or TTSS. The residual analyses, however, suggested a different picture. Although CD9⁺CD14⁺ sEV levels were associated with established measures of thoracic injury severity, elevated EV levels relative to those predicted by AIS Chest or TTSS remained associated with unfavorable respiratory-support trajectories. Patients with higher-than-expected CD9⁺CD14⁺ levels demonstrated substantially increased rates of tracheostomy and prolonged mechanical ventilation compared with patients whose EV levels were consistent with their anatomical injury severity. These exploratory findings suggest that thoracic injury scores and EV-associated information may capture related but non-identical aspects of the post-traumatic response. This observation was most consistent following adjustment for AIS Chest, whereas similar trends after TTSS adjustment did not reach statistical significance.

What might explain this apparent divergence between injury severity scores and EV-associated information? Although the present analyses cannot establish biological mechanisms, they provide a conceptual framework for interpreting the observed findings. Our exploratory results are consistent with the hypothesis that AIS Chest and TTSS primarily describe the extent of anatomical thoracic injury, whereas CD9⁺CD14⁺ sEVs may reflect aspects of the early biological response to that injury. These complementary information layers therefore may provide different, yet clinically relevant, perspectives on the post-traumatic response. From this perspective, EVs should not be viewed as replacements for established injury scores but rather as exploratory biomarkers that may complement conventional anatomical assessment by providing additional insight into the host response following trauma.

The biological interpretation of CD9⁺CD14⁺ sEVs is also supported by current understanding of monocyte and macrophage biology. Importantly, the observed increase in CD9⁺CD14⁺ sEVs should not be interpreted as being unique to thoracic trauma. Rather, extracellular vesicle release represents a fundamental biological response to cellular activation, tissue injury, and inflammation that has been described across a broad spectrum of traumatic and inflammatory conditions. Similar alterations in circulating EV profiles have also been reported in systemic disorders such as sepsis and hypertension or sepsis in trauma, further supporting the concept that EVs reflect general inflammatory and cellular activation pathways rather than disease-specific mechanisms [34, 35].

Nevertheless, monocytes and macrophages play central roles in trauma-associated inflammation and lung injury. Experimental studies have demonstrated that modulation of CD14-dependent pathways can influence both local and systemic inflammatory responses following blunt thoracic trauma [36]. Following injury, circulating monocytes are rapidly recruited and differentiate into macrophages, contributing to both pulmonary and systemic inflammatory responses [37, 38]. Thoracic trauma, however, represents a particularly relevant clinical setting because direct pulmonary injury, disruption of the alveolar-capillary barrier, and early recruitment of monocytes and macrophages are closely linked to subsequent respiratory-support requirements. Trauma may disrupt normal maturation pathways and promote pro-inflammatory phenotypes associated with acute lung injury [13, 39]. Elevated CD14⁺ EV levels may therefore reflect the combined effects of cellular activation, recruitment, apoptosis, and turnover within the monocyte–macrophage compartment [16, 40]. Clinical studies further support this interpretation by demonstrating associations between monocyte dysfunction and adverse outcomes in trauma and lung disease [41–43]. At the same time, abdominal trauma, pelvic trauma, and other severe injuries are likewise capable of inducing extracellular vesicle release through systemic inflammatory pathways. Therefore, the present findings should be interpreted as identifying a biologically plausible EV signature within thoracic trauma rather than implying thoracic specificity of EV release itself. Future comparative studies across different trauma patterns will be required to determine whether the observed CD9⁺CD14⁺ EV profile reflects a thoracic-specific biological response or a more general systemic response to severe injury. Nevertheless, our study cannot establish causality, and mechanistic studies are required to further clarify the biological origin and functional relevance of these EV populations.

While our primary interpretation focuses on monocyte-associated CD14⁺ EVs, the contribution of CD61⁺ EVs should also be acknowledged. Although CD61 is not exclusively platelet-specific, it is commonly used to capture platelet-associated vesicles in trauma and coagulation research [10, 44, 45]. Platelets play a fundamental role in hemostasis, vascular integrity, and wound healing and release EVs upon activation [43]. The observed associations of CD9⁺CD61⁺ EVs with respiratory-support trajectories may therefore reflect platelet activation and vascular responses following injury, although their predictive contribution appeared smaller than that observed for CD14⁺ EVs.

Several limitations should be considered. First, our cohort size was relatively small and reflects a single-center experience, limiting generalizability. In addition, the relatively small number of tracheostomy events (*n* = 14) limited the robustness of multivariable modelling. Consequently, the preliminary machine-learning and AUROC analyses should be interpreted as exploratory comparisons of predefined biomarker, clinical injury, laboratory, and oxygenation information layers rather than as the development or validation of a definitive clinical prediction model, and some degree of overfitting cannot be excluded. Second, the exploratory design precludes definitive conclusions regarding clinical implementation, and the findings should be regarded as hypothesis-generating. Third, despite the use of repeated stratified three-fold cross-validation for internal performance estimation, this approach does not replace external validation and may still yield optimistic performance estimates in a small exploratory cohort. External validation in larger independent cohorts therefore remains essential. Furthermore, EV sampling was standardized to 24 h after hospital admission rather than 24 h after injury. As injury-to-admission intervals were not consistently available, we could not assess the influence of sampling time relative to injury on circulating EV levels. Future prospective studies incorporating precisely documented injury and sampling times should investigate the temporal kinetics of EV release following thoracic trauma. Thus, the investigated respiratory-support endpoints—including tracheostomy, prolonged mechanical ventilation, and prolonged ICU stay—represent closely related manifestations of an unfavorable respiratory-support trajectory rather than fully independent clinical outcomes and should therefore be interpreted within this clinical context.

Finally, although clinically relevant variables were included, unmeasured confounders and variability in trauma patterns may have influenced the observed associations. In particular, prolonged ventilation, ICU duration, and tracheostomy are not exclusively determined by thoracic trauma but may also be influenced by traumatic brain injury, abdominal injury, pelvic injury, sedation requirements, airway protection, institutional weaning strategies, center-specific clinical practice, and other components of overall trauma burden. In addition, although CD14 and CD61 are well-established markers of monocyte- and platelet-associated extracellular vesicles, respectively, surface-marker profiling alone does not definitively establish the precise cellular origin of individual vesicles. Therefore, the biological interpretation of these EV populations should be regarded as biologically plausible but not definitive. A further limitation is the absence of quantitative imaging-based assessment of pulmonary contusion burden. Future studies integrating EV profiling with standardized CT-based lung injury quantification may help clarify the relationship between biological response and structural pulmonary damage.

## Conclusion

In summary, our exploratory study provides first evidence that somewhat experimental CD9⁺CD14⁺ small extracellular vesicles are associated with unfavorable respiratory-support trajectories following thoracic trauma. Within this exploratory cohort, CD9⁺CD14⁺ sEVs demonstrated reasonable predictive performance comparable to established clinical injury scores and superior to laboratory- and oxygenation-based information layers for several clinically relevant endpoints. Residual analyses further suggest that EV-associated information may capture aspects of the biological host response not fully reflected by anatomical injury severity alone. Together, these exploratory findings support our working hypothesis in which injury severity scores characterize the extent of anatomical damage, whereas EV-based biomarkers may provide insight into the biological response to injury. Larger multicenter studies are warranted to validate these observations and to explore integration of exploratory EV-associated biomarkers into future trauma risk stratification approaches.

## Supplementary Information


Supplementary Material 1.


## Data Availability

The datasets analyzed during the current study are derived from an EV dataset previously published by our group (Frontiers in Immunology, 2023) [10] and have been reprocessed addressing novel questions on the establishment of tracheostomy and ventilation in thorax trauma due to prolonged weaning for this analysis. All Python code used for the prediction, and visualization—as well as example input files—will be made available to qualified researchers upon reasonable request to the corresponding author.

## Abbreviations

AIS: Abbreviated Injury Scale
ALI: Acute Lung Injury
ASA: American Society of Anesthesiologists (physical status classification)
BMI: Body Mass Index
CD14: Cluster of Differentiation 14 (monocyte marker)
CD61: Cluster of Differentiation 61 (platelet marker)
CD9: Cluster of Differentiation 9 (tetraspanin marker)
CI: Confidence Interval
CRP: C-reactive Protein
CT: Computed Tomography
EGFR: Epidermal Growth Factor Receptor
EV: Extracellular Vesicle
ICU: Intensive Care Unit
IM: Intermediate Monocyte
ISS: Injury Severity Score
LOFS: Lung Organ Failure Score
LOS: Length of stay
MISeV2018: Minimal Information for Studies of Extracellular Vesicles 2018
NCM: Non-Classical Monocyte
NISS: New Injury Severity Score
PaO₂/FiO₂: Ratio Arterial Oxygen Partial Pressure / Fraction of Inspired Oxygen
PCA: Principal Component Analysis
PCT: Procalcitonin
R²: Coefficient of Determination
RAGE: Receptor for Advanced Glycation Endproducts
RTS: Revised Trauma Score
sEV: small Extracellular Vesicle
SD: Standard Deviation
T3P-Score: Tracheostomy in Thoracic Trauma Prediction Score
TBI: Traumatic Brain Injury
TTSS: Thoracic Trauma Severity Score
TRISS: Trauma and Injury Severity Score
