# Supplementary material for: Beyond injury severity: monocyte-associated small extracellular vesicles distinguish respiratory-support trajectories following thoracic trauma
Source: BMC Pulm Med. 2026 Aug 31;26:383. doi: 10.1186/s12890-026-04653-w (PMC13528151; doi:10.1186/s12890-026-04653-w)
Supplement: Supplementary file 1 [file 12890_2026_4653_MOESM1_ESM.docx]

Supplementary Figures and Tables

Supplementary Figure S1 AUROC heat map across clinical endpoints and information layers. Heat map summarizing the predictive performance of EV-associated biomarkers, injury severity scores, laboratory parameters, and oxygenation-based models across all investigated clinical endpoints. Cells display mean cross-validated AUROC values ± standard deviation obtained from repeated stratified cross-validation (3-fold, 30 repeats). Warmer colors indicate higher predictive performance, whereas cooler colors indicate lower predictive performance. Information layers included CD9⁺CD14⁺ and CD9⁺CD61⁺ small extracellular vesicles (sEVs), a clinical injury model (age, ISS, AIS chest, RibScore, pneumothorax, hemothorax, abdominal injury, pelvic injury, and traumatic brain injury), a laboratory model (white blood cell count, platelets, CRP, IL-6, and albumin), and PaO₂/FiO₂ ratios on Day 0 and Day 1 after exclusion of patients with traumatic brain injury. Clinical endpoints included predefined respiratory-support trajectories (tracheostomy, prolonged mechanical ventilation >5 days, prolonged ICU stay >5 days), sensitivity endpoints (>10 days), exploratory respiratory failure endpoints, and pneumonia. The heat map provides a global overview of model performance across all investigated outcome measures.

Supplementary Figure S2 Sensitivity analysis of severe respiratory-support trajectories using >10-day thresholds. Cross-validated receiver operating characteristic (ROC) curves comparing the predictive performance of early information layers for severe respiratory-support trajectories defined as prolonged mechanical ventilation (>10 days) and prolonged ICU stay (>10 days). Displayed models include CD9⁺CD14⁺ small extracellular vesicles (sEVs), the clinical injury model, and PaO₂/FiO₂ ratios on Day 0 and Day 1 after exclusion of patients with traumatic brain injury. Mean AUROC values were derived from repeated stratified cross-validation (3-fold, 30 repeats). The diagonal dashed line indicates random classification performance (AUROC = 0.5). These analyses were performed as sensitivity analyses using a stricter definition of prolonged respiratory-support trajectories than the predefined primary threshold of >5 days.

Supplementary Table S2a Predictive performance of early information layers for severe respiratory-support trajectories (>10 days). Mean AUROC ± standard deviation obtained from repeated stratified cross-validation (3-fold, 30 repeats) for EV-associated biomarkers, the clinical injury model, the laboratory model, and PaO₂/FiO₂-based models. Severe respiratory-support trajectories were defined as prolonged mechanical ventilation exceeding 10 days (Vent >10 d) and prolonged ICU stay exceeding 10 days (ICU >10 d). PaO₂/FiO₂ Day 0 and Day 1 analyses were performed after exclusion of patients with traumatic brain injury to reduce confounding from early intubation and ventilation-related interventions.
Endpoint
CD9+CD14+
CD9+CD61+
Clinical injury
Laboratory model
PaO2/FiO2 D0
PaO2/FiO2 D1
Vent >10 d
0.808 ± 0.115
0.726 ± 0.132
0.741 ± 0.111
0.359 ± 0.116
0.420 ± 0.171
0.426 ± 0.150
ICU >10 d
0.745 ± 0.107
0.704 ± 0.119
0.834 ± 0.094
0.557 ± 0.120
0.425 ± 0.129
0.519 ± 0.147

Supplementary Table S2b Pairwise comparisons of predictive performance for severe respiratory-support trajectories (>10 days). Pairwise comparisons of AUROC distributions obtained from repeated stratified cross-validation were performed using Wilcoxon signed-rank tests. Holm correction was applied to adjust for multiple testing. The table focuses on predefined comparisons involving CD9⁺CD14⁺ sEVs versus CD9⁺CD61⁺ sEVs, the clinical injury model, and the laboratory model. Raw and Holm-adjusted p-values are reported.
Endpoint
Comparison
p_raw
p_holm
p_raw_formatted
p_holm_formatted
Vent >10 d
CD9+CD14+ vs CD9+CD61+
2.793061 x 10-9
1.675836 x 10-8
<0.001
<0.001
Vent >10 d
CD9+CD14+ vs Clinical injury
3.100773 x 10-4
1.550387 x 10-3
<0.001
0.002
Vent >10 d
CD9+CD14+ vs Laboratory model
1.729873 x 10-16
2.594809 x 10-15
<0.001
<0.001
ICU >10 d
CD9+CD14+ vs CD9+CD61+
1.200930 x 10-3
2.401859 x 10-3
0.001
0.002
ICU >10 d
CD9+CD14+ vs Clinical injury
1.393303 x 10-7
4.179908 x 10-7
<0.001
<0.001
ICU >10 d
CD9+CD14+ vs Laboratory model
6.232646 x 10-14
5.609381 x 10-13
<0.001
<0.001
